# Alterations of Rice (*Oryza sativa* L.) DNA Methylation Patterns Associated with Gene Expression in Response to Rice Black Streaked Dwarf Virus

**DOI:** 10.3390/ijms21165753

**Published:** 2020-08-11

**Authors:** Linying Li, Yuqing He, Xueying Zhang, Hehong Zhang, Zongtao Sun, Junmin Li, Gaojie Hong

**Affiliations:** 1 College of Horticulture and Plant Protection, Yangzhou University, Yangzhou 225009, China; anran6297@163.com; 2 State Key Laboratory for Managing Biotic and Chemical Threats to the Quality and Safety of Agro-Products Institute of Virology and Biotechnology, Zhejiang Academy of Agricultural Sciences, 198 Shiqiao Road, Hangzhou 310021, China; yqhe_123@126.com (Y.H.); zhangxuey89@163.com (X.Z.); 3 The State Key Laboratory Breeding Base for Sustainable Control of Pests and Disease, Institute of Plant Virology, Ningbo University, Ningbo 315211, China; 18758878079@163.com (H.Z.); sunzongtao@nbu.edu.cn (Z.S.); zjlijunmin@126.com (J.L.)

**Keywords:** DNA methylation, rice, RBSDV, differentially methylated genes, gene expression

## Abstract

Rice black-streaked dwarf virus (RBSDV) causes severe yield losses in rice (*Oryza sativa* L.) in China. Studies have shown that the mechanisms of DNA methylation-mediated plant defense against DNA viruses and RNA viruses are different. However, in rice its function in response to infection of RBSDV, a double-stranded RNA virus, remains unclear. In this study, high-throughput single-base resolution bisulfite sequencing (BS-Seq) was carried out to analyze the distribution pattern and characteristics of cytosine methylation in RBSDV-infected rice. Widespread differences were identified in CG and non-CG contexts between the RBSDV-infected and RBSDV-free rice. We identified a large number of differentially methylated regions (DMRs) along the genome of RBSDV-infected rice. Additionally, the transcriptome sequencing analysis obtained 1119 differentially expressed genes (DEGs). Correlation analysis of DMRs-related genes (DMGs) and DEGs filtered 102 genes with positive correlation and 71 genes with negative correlation between methylation level at promoter regions and gene expression. Key genes associated with maintaining DNA methylation in rice were analyzed by RT-qPCR and indicated that *OsDMT702* might be responsible for the global increase of DNA methylation level in rice under RBSDV stress. Our results suggest important roles of rice DNA methylation in response to RBSDV and provide potential target genes for rice antiviral immunity.

## 1. Introduction

DNA methylation, a conserved epigenetic modification involved with the addition of a methyl group to cytosines, is important for plant genome stability, environmental responses and developmental regulation [1]. Plant DNA methylation occurs on the cytosine bases in CG, CHG or CHH contexts (H represents A, T, or C) by conserved DNA methyltransferases [2]. DNA methylation is regulated and maintained by two independent pathways: the de novo methylation pathway and the maintenance methylation pathway [3,4], whereas active plant DNA demethylation is associated with a base excision repair pathway [2]. *Methyltransferase 1* (*MET1*) is responsible for the maintenance of CG methylation, while *chromomethylase 3* (*CMT3*) or *CMT2* plays a leading role in the catalyzing CHG methylation [5,6,7]. *CMT2* and domain rearranged methyltransferases (*DRM1* and *DRM2*) maintain the methylation of CHH contexts through persistent de novo methylation in which *RNA polymerase IV* (Pol IV), *RNA-dependent RNA polymerase 2* (*RDR2*), *Dicer-like 3* (*DCL3*) and Argonaute 4 (*AGO4*) or *AGO6* are required [8]. DNA demethylation enzymes, including *REPRESSOR OF SILENCING 1* (*ROS1*), *DEMETER* (*DME*), *DEMETER-LIKE 2* (*DML2*), and *DML3* in *Arabidopsis*, can actively erase DNA methylation through a base excision repair mechanism [9].

Recent studies have provided evidence that DNA methylation modulates immune responses against plant viruses. The chromatin-like organisation of plant DNA viruses is more easily methylated to restrict their proliferation due to their single-stranded DNA (ssDNA) genome and double-stranded DNA (dsDNA) replication process [10]. Thus, viral genome methylation can be employed by plants as an epigenetic defense against DNA viruses. Tomato *Ty-1*, an RNA-dependent RNA polymerase from *Solanum chilense*, confers resistance to tomato yellow leaf curl virus (TYLCV) by increasing cytosine methylation of viral genomes [11]. Silencing of *Nicotiana benthamiana histone deacetylase 6* (*NbHDA6*) expression results in a reduced DNA methylation level of the viral genome and enhanced host susceptibility to TYLCV infection [12]. Unlike DNA viruses, RNA viruses possess scarcely methylated RNA genomes for replication [10]. DNA methylation regulates the expression of plant disease resistance genes to respond to the infection of RNA viruses [13,14,15]. For example, DNA (de)methylation altered the susceptibility/resistance of *Arabidopsis* plants to tobacco rattle virus (TRV) infection by regulating the expression of disease resistance genes [16]. Alfalfa mosaic virus (AMV) infection is controlled by m^6^A modification of its genomic RNAs through the interaction between viral CP and *At*ALKBH9B, an *Arabidopsis thaliana* protein with ssRNA m^6^A demethylase activity [17]. This reseach indicates that DNA methylation plays a dominant role in antiviral defense.

Rice black-streaked dwarf virus (RBSDV), a plant-infecting agent with ten segments of double-stranded RNA (dsRNA), has caused dramatic losses in rice [18]. RBSDV is transmitted to the plants by the small brown planthopper (SBPH, *Laodelphax striatellus*) in a circulative and persistent manner [19]. RBSDV-infected rice plants typically display symptoms, such as leaf darkening and plant stunting or black-streaked galls along the veins [20]. Previous studies on rice plants defense against RBSDV mainly focused on RNAi [21,22] and plant hormone signaling pathways [23]. For instance, the jasmonate (JA) and auxin signaling pathways enhance plant defense against RBSDV [24,25], whereas brassinosteroid (BR) and abscicic acid (ABA) pathways facilitated the viral infection [25,26]. However, the role of host DNA methylation in RBSDV infection still remains unclear.

In this study, single-base resolution bisulfite sequencing (BS-Seq) and RNA sequencing (RNA-Seq) were used to identify the pattern changes of cytosine methylation and their correlation with gene expression in RBSDV-infected rice plants. Specific methylation analyses showed that the general DNA methylation level of rice increased notably during RBSDV infection. We found that many differentially methylated genes (DMGs) under RBSDV infection were also transcriptionally regulated. In summary, our study reveals the dynamics of DNA methylation in rice infected by RBSDV and suggests a new mechanism of non-natural immunity against virus infection.

## 2. Results

### 2.1. Genome-Wide Mapping of DNA Methylome Variation in Rice Infected by RBSDV

Whole-genome bisulfite sequencing (WGBS) of *O. sativa* L. *japonica Nipponbare* (NIP) inoculated with RBDSV was carried out to investigate the role of DNA methylation in the rice response to viral infection. WGBS generated 85,333,334, 84,048,720, and 88,499,894 raw reads from three virus-free NIP samples, and 93,403,218, 90,424,096 and 90,666,668 raw reads from three RBSDV-infected NIP samples. After quality assessment and data cleanup 82,416,848, 81,447,850 and 85,564,338 valid data of virus-free samples (mock), and 90,367,002, 87,853,866 and 88,001,962 valid data of RBSDV-infected samples (RB) with more than 94% Q30 bases were filtered, respectively. The average GC percentage of 6 rice libraries was 29.14% (Table S1). Of those valid data, 75.74% (mock-1), 76.81 (mock-2), 76.38% (mock-3), 77.40% (RB-1), 76.95% (RB-2) and 77.47% (RB-3) were uniquely mapped to the genome of rice (Table S1). The coverage and depth of this study were comparable to that of rice exposed to cadmium (Cd) (Figure S1A) [27]. These results were reliable for subsequent high-quality genome-wide methylation analysis.

### 2.2. Differential Landscapes of DNA Methylation Marks in Virus-Free and RBSDV-Infected Rice

We identified 443,719,059, 411,439,030 and 458,827,065 methylcytosines (mCs) from all map reads of virus-free rice, and 505,085,616, 551,201,939 and 491,635,813 mCs from all map reads of RBSDV-infected rice, respectively. Of these, approximately, 51%–53% mCs occurred at CG sites and 49%–47% at non-CG sites (21%–23% at CHH and 26%–27% at CHG; H=A, T, or C) in virus-free rice. Infection of RBSDV led to a slight drop of genomic methylation degree at CG sites, the mC counts (total mCs) at mCHHs was higher under RBSDV stress, and there was no obvious difference at CHG sites, suggesting that DNA methylation in CG, CHG, and CHH sequence contexts was unevenly affected by RBSDV (Figure S1B). 

**Figure 1:**
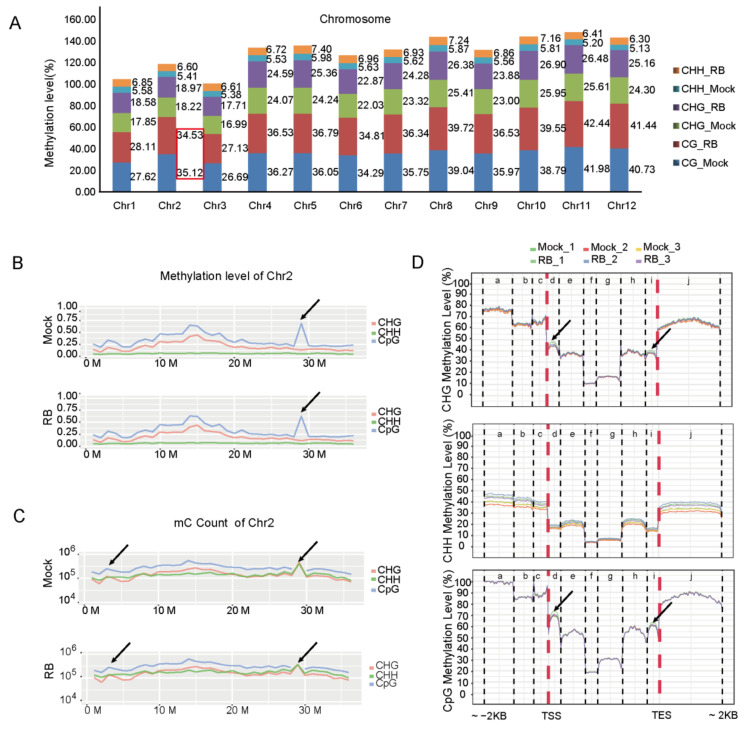
Differential CG and non-CG methylation levels in genomic regions of virus-free or RBSDV-infected rice. (**A**) Levels of CG methylation and non-CG methylation in rice chromosomes. Methylation level: percentage of reads showing mC among all the reads covering the same cytosine site. (**B**,**C**) The density profiling of methylcytosines (**B**) and mC count (**C**) in chromosome 2. Smoothed lines represent the density (CpG, CHG and CHH) in each context. Black arrows highlight the differences between the Virus-free and RBSDV-infected rice chromosome 2. One biological replicate was shown. Density profiling: mC per million base pairs. (**D**) DNA methylation levels of gene body and promoter regions (a: Distal promoter. b: Intermediate promoter. c: Proximal promoter. d: First exon. e: First intron. f: Internal exon. g: Internal intron. h: Last intron. i: Last exon. j: Downstream). Methylation levels of CpG, CHG, and CHH are shown. Transcription start site (TSS) and transcription end site (TES) are indicated.

DNA methylation levels throughout the 12 chromosomes were further reviewed. However, the entire chromosomal methylcytosine levels at CG, CHG and CHH site were higher in RBSDV-infected rice than in the controls (virus-free) except the CG site of chromosomes 2 (Chr2) under RBSDV stress (Figure 1A,B). The DNA methylation patterns of virus-free and RBSDV-infected rice were similar (Figure S2), whereas the density profile of methylcytosines in Chr2 of rice was clearly distinguished (Figure 1C). The average methylcytosine levels of gene body and promoter regions were further inspected in different regions of promoter, exon, intron, and downstream sequences, and it was found that, under RBSDV stress, CHG and CG methylation were increased in first exon and last exon regions, respectively. Further analysis showed that the CHH methylation levels in all specific regions were higher in RBSDV-infected rice than in the control (Figure 1D).

### 2.3. Differentially Methylated Regions (DMRs) Identified in Rice

**Figure 2:**
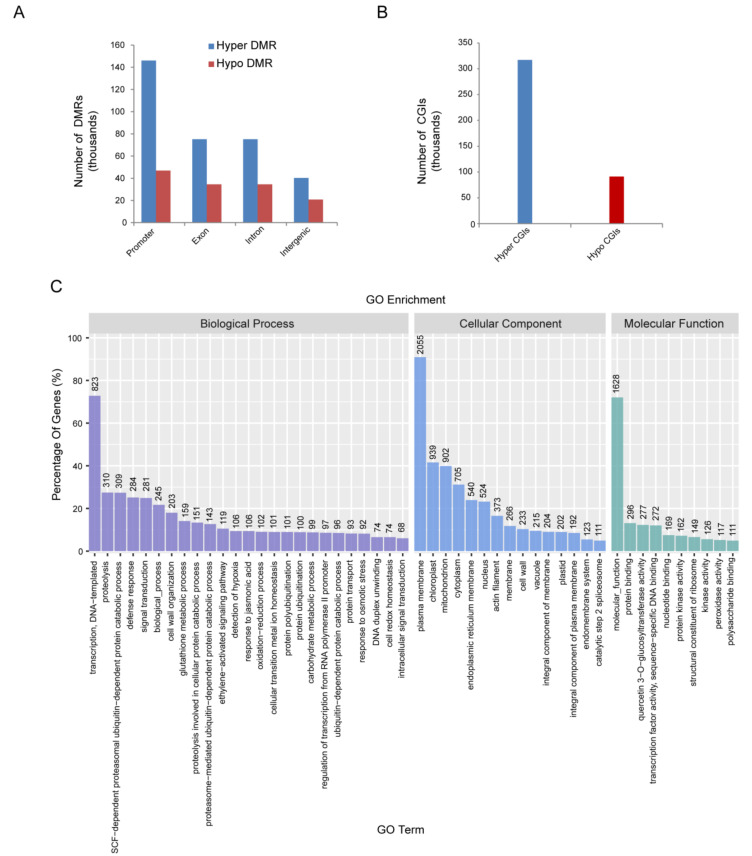
Characterization of DMR–associated genes. (**A**,**B**) Differentially methylated regions (DMRs) number of gene body (**A**) and CGI (**B**) identified in rice infected by RBSDV. Promoter region is defined as −1 kb to +1 kb flanking transcription start site (TSS); gene body refers to +1 kb downstream of TSS to transcription end site (TES), and the remainder is considered as intergenic region. (**C**) GO analysis of DMR-associated genes based on their functional enrichment.

The genomic regions associated with CG, CHG and CHH hypermethylation or hypomethylation were profiled to investigate specific DNA methylation in RBSDV-infected rice. A total of 980,913 non-redundant RBSDV-responsive DMRs were examined (*p* < 0.05), among which 734,234 were hypermethylated (hyper-DMRs) and 246,679 were hypomethylated (hypo-DMRs). Further analysis showed that DMRs in promoter regions were higher compared to the exon, intron, and intergenic regions (Figure 2A). Methylation of CpG islands (CGIs) at gene promoters has been reported to be associated with the silencing of numerous genes affecting a variety of vital cellular processes [28,29]. We observed a total of 408,352 CGIs (317 053 hypermethylated and 91,299 hypomethylated, respectively) (Figure 2B). There were more hypermethylated genes than hypomethylated genes which was consistent with the previous studies [15]. The DMR-associated genes obtained according to differential methylation statistics in 2000 regions upstream of the transcription start site of the gene were classified into three groups according to biological process, cellular component and molecular function based on rice GO annotation information. High clusters pointed to transcription, DNA-templated as biological process, plasma membrane as cellular component, and molecular function as the molecular function group (Figure 2C).

### 2.4. Transcriptomic Analysis of Genes Associated with RBSDV Infection in Rice

**Figure 3:**
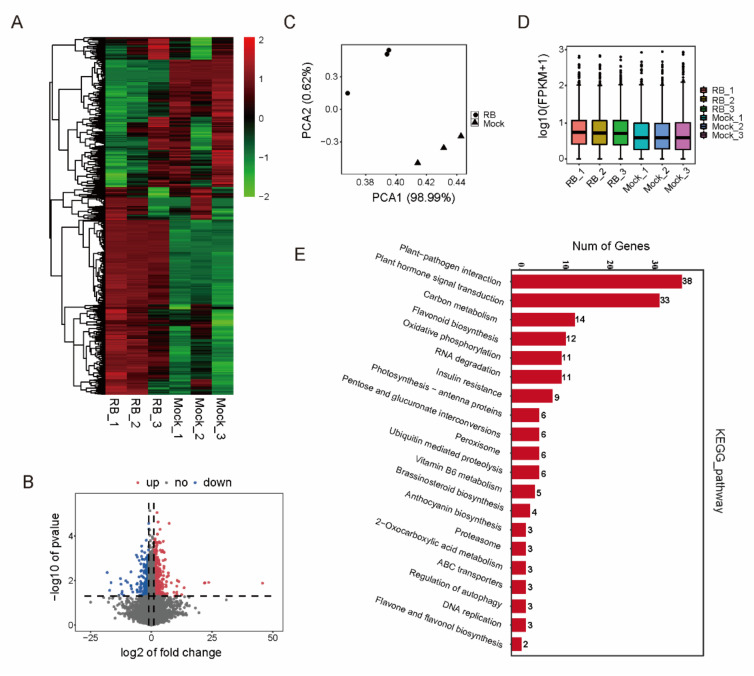
Analysis of RBSDV-infected rice transcriptome. (**A**) Heatmap representation of a one-dimensional hierarchical clustering of differential gene expression as determined by mRNA-seq for the RBSDV-infected rice relative to the control (RBSDV-free). (**B**) Differential transcript abundance of RBSDV-infected and RBSDV-free rice. The y-axis represents the *p*-value relative to the change in the mean normalized expression of all transcripts (x-axis). Red dots indicate the up-regulated genes, blue dots indicate the down-regulated genes (*p* < 0.01). (**C**) Results of principal component analysis. DEGs of RBSDV-infected rice had visible differences. (**D**) Box-whisker Plot FPKM (fragments per kilobase of exon per million fragments mapped) of DEGs (three biological replicates of RBSDV-infected and RBSDV-free rice, respectively). (**E**) KEGG enrichment analysis of RBSDV-infected and control rice transcripts. Y-axis represents pathways, while x-axis represents gene number (*p* < 0.05).

Although a number of genes indicated previously were modified by DNA methylation in RBSDV-inoculated plants, whether they were transcriptionally affected by RBSDV was unknown. A genome-wide analysis of transcripts was performed to identify the genes that were differentially methylated and expressed from RBSDV-infected plants using the high throughput RNA-Seq technology. The transcript abundance was assessed from RBSDV-free and RBSDV-infected rice seedlings. A total of 1119 differentially expressed genes (DEGs) (|log2foldchange| ≥ 1, *p* < 0.05) under RBSDV infection were identified. RBSDV induced overall changes in gene expression (Figure 3A,B). DEGs in three biological replicates were similar, confirming the reliability of the data (Figure 3C,D). 181 DEGs were assigned to the 20 canonical reference pathways in the Kyoto Encyclopedia of Genes and Genomes (KEGG) that may play important roles in plants responses to various viruses [15]. 38 DEGs participated in plant-pathogen interactions and 33 DEGs were involved in plant hormone signal transduction (Figure 3E).

### 2.5. Correlation between DNA Methylation and Gene Expression during RBSDV Infection

**Figure 4:**
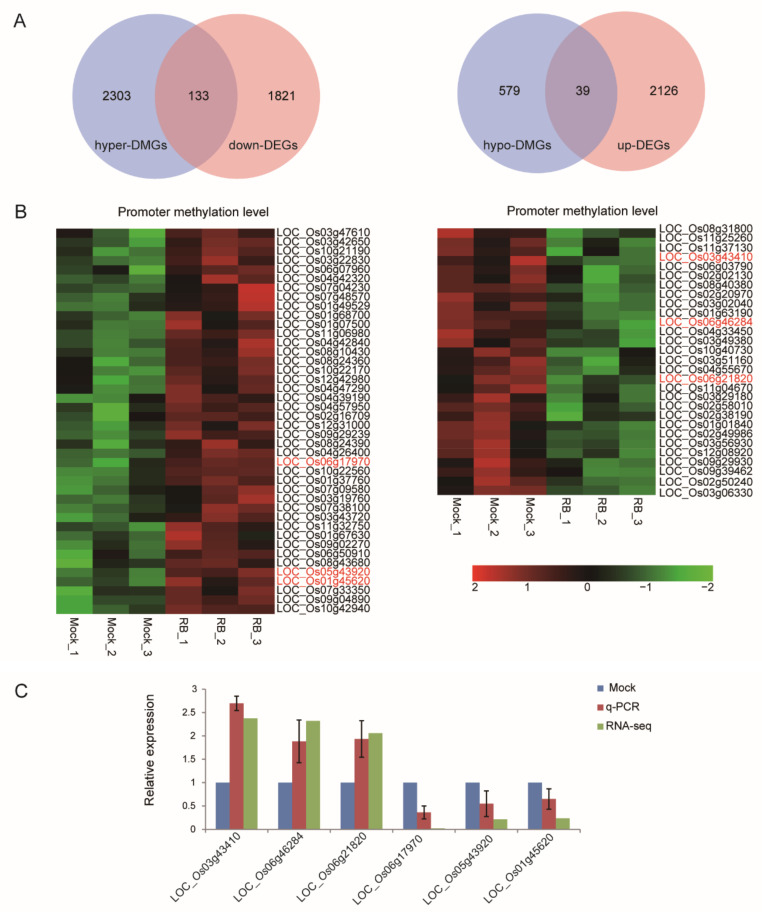
Combinational analysis of genes that changed in DNA methylation and transcriptional expression. (**A**) Venn diagrams display the negative association between DNA methylation region (DMR)-associated genes (*p* < 0.05) and differentially expressed genes (*p* < 0.05) induced by RBSDV. (**B**) Heatmap represents the methylation levels of 71 RBSDV-responsive genes. (**C**) RT-qPCR validated six randomly selected RBSDV-responsive mRNAs from up-regulated and down-regulated candidate genes. RT-qPCR results were normalized to the data from RBSDV-free leaves, respectively. Vertical bars represent standard deviation (SD) of the mean of five biological replicates.

Modulation of DNA methylation in promoters represents a potential mechanism to adjust plant gene expression following exposure to different stresses [30]. To maximize genes regulated by the methylation on promoter regions, the threshold of DMRs-associated genes and DEGs was adjusted to a *p*-value < 0.05. We identified 2964 promoter DMR-associated genes (2436 hyper-DMGs, 618 hypo-DMGs) and 4118 DEGs (2164 up-regulated, 1954 down-regulated). Cross analysis of DMGs and DEGs showed that 125 hyper-DMGs were up-regulated at the transcriptional level and 26 hypo-DMGs were down-regulated (Figure S4A), whereas 39 hypo-DMGs were up-regulated at the transcriptional level and 133 hyper-DMGs were down-regulated (Figure 4A). We limited to DEGs with a 1.5-fold change of the up-regulated genes and 2-fold change of the down-regulated genes, allowing only 102/71 genes with positive/negative correlation between DMGs and DEGs upon RBSDV infection to be filtered (Supplementary Data S1 and Supplementary Data S2). The heatmap revealed a different DNA methylation level in promoter regions of 102 genes (87 hyper-up, 15 hypo-down) with positive correlation (Figure S4B) and 71 genes (29 hypo-up, 42 hyper-down) with negative correlation between DMGs and DEGs (Figure 4B). Many studies focus on the negative correlation between DNA methylation status in promoters and gene expression in plant for its crucial roles during different growth stages and under stress conditions [28,31,32]. Many of the 71 genes were involved in biosynthesis of amino acids, plant-pathogen interaction, plant hormone signal transduction and protein processing in endoplasmic reticulum pathways (Figure S3). Accordingly, six representative genes for four KEGG pathways were further selected for transcript analysis by RT-qPCR. Among these were, LOC_Os03g43410 (OsIAA12-Auxin-responsive Aux/IAA gene family member, expressed), LOC_Os05g43920 (auxin response factor 14, putative, expressed) and LOC_Os01g45620 (CGMC_MAPKCMGC_2.5-CGMC includes CDA, MAPK, GSK3, and CLKC kinases, expressed) involved in KEGG pathways of Plant hormone signal transduction; LOC_Os06g17970 (NBS-LRR disease resistance protein, putative, expressed) involved in KEGG pathway of Plant-pathogen interaction; LOC_Os06g46284 (glycosyl hydrolase, family 31, putative, expressed) involved in KEGG pathway of Galactose metabolism; and LOC_Os06g21820 (jasmonate O-methyltransferase, putative, expressed) involved in KEGG pathway of α-linolenic acid metabolism. LOC_Os03g43410, LOC_Os06g46284 and LOC_Os06g21820 were hypomethylated and up-regulated at the transcriptional level, while LOC_Os06g17970, LOC_Os05g43920 and LOC_Os01g45620 were hypermethylated and down-regulated. RT-qPCR results were consistent with RNA-seq data (Figure 4C). All of them showed a negative correlation between methylation level and gene expression, indicating that these genes may be modified by DNA methylation.

### 2.6. Expression of Genes Related to DNA Methylation in RBSDV-Infected Rice

Recent studies have identified 10 DNA methyltransferases (DMT) family members responsible for DNA methylation in rice. *OsDMT702* (*LOC_Os03g58400, OsMET1-1*) and *OsDMT707* (*LOC_Os07g08500, OsMET1-2*) are responsible for maintaining CpG methylation [33]. *OsDMT701* (*Os03g12570*), *OsDMT703* (*Os05g13790*) and *OsDMT704* (*Os10g01570*) play a critical role in maintaining CHG methylation [6,27]. *OsDMT705* (*Os01g42630*), *OsDMT706* (*Os03g02010*), *OsDMT708* (*Os12g01800*), *OsDMT709* (*Os11g01810*) and *OsDMT710* (*Os05g04330*) are methyltransferases responsible for DNA CHH-specific methylation [34,35]. 

**Figure 5:**
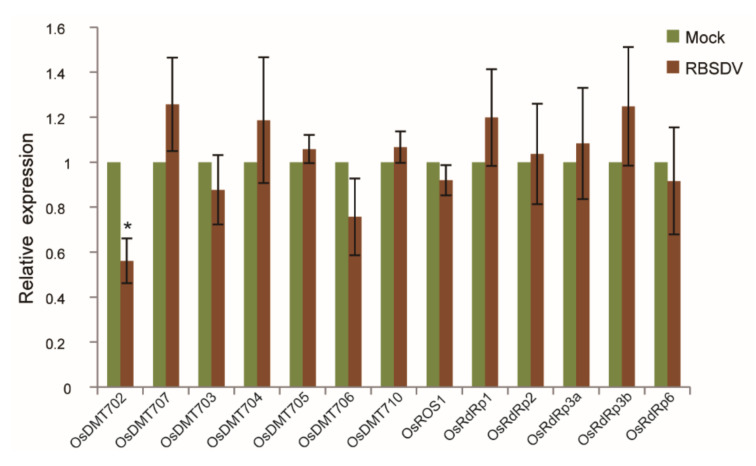
Expression levels of genes involved in DNA methylation/demethylation in rice infected by RBSDV. Each biological sample with 8 to 12 RBSDV-infected rice seedlings at 30 dpi were used for RT-qPCR. Vertical bars represent standard deviation (SD) of the mean of five biological replicates. dpi: days post inoculation. Mock: RBSDV-free seedling. Significant differences were identified using two-tailed Student’s *t*-test. * At the top of columns indicates significant difference at *p* ≤ 0.05.

In addition, five RNA-dependent RNA polymerase (RDR) protein genes, including *OsRDR1* (*Os02g50330*), *OsRDR2* (*Os04g39160*), *OsRDR3a* (*Os01g10130*), *OsRDR3b* (*Os01g10140*) and *OsRDR6* (*Os01g34350*) which are required for RNA-directed DNA methylation, have been identified in rice [36]. RT-qPCR was performed to analyze their expression abundance under RBSDV stress. The expression level of *OsDMT701*, *OsDMT708* and *OsDMT709* were too low to be detected. Expression of *OsDMT702* was significantly repressed in RBSDV-infected rice, while expression of *OsDMT704*, *OsDMT707*, *OsRDR1* and *OsRDR6* was weakly enhanced. *OsDMT706* was slightly reduced, and no significant change was detected in the expression level of the remaining genes (Figure 5). These results indicate that RBSDV altered the expression of some key genes maintaining DNA methylation of rice to different extent.

## 3. Discussion

DNA methylation was proposed to be a new modulator of the plant response to biotic and abiotic stresses [9,37,38]. Plant genome methylation and viral methylation has been reported to be a new layer of epigenetic change in response to viral infection [39]. Methylation across genome of DNA viruses restricts their replication, unlike RNA viruses that do not use a DNA replication process [10]. RBSDV is a member of the genus *Fijivirus* with dsRNA as genome for replication, its infection results in severe yield losses in rice [40]. Most of the work on DNA methylation of rice under stress conditions mainly focused on chilling, pesticide, drought and salinity stresses [35,41,42]. However, analysis of DNA methylation changes of rice in response to viruses, such as RBSDV, has not been reported. We profiled DNA methylomes of rice seedlings infected by RBSDV through whole-genomic bisulfite sequencing. Although overall genomic cytosine methylation levels of the RBSDV-infected and RBSDV-free rice plants are similar, we discovered local variations for the density profile at CG, CHG and CHH sites. Genomes of NIP infected by RBSDV undergo a global increase in DNA methylation, which is consistent with changes induced by pesticide atrazine and low-temperature [35,42], but is contrary to NIP exposed to Cd [27]. The CG, CHG and CHH contexts in TRV-infected *Arabidopsis* were slightly hypomethylated compared to mock-inoculated controls [16]. These results suggest that the function of DNA methylation in plants is different under diverse stresses. The increased cytosine methylation level in the CHH context is common to rice infected by RBSDV or *Citrullus lanatus* infected by cucumber green mottle mosaic virus (CGMMV) [15], which further indicates the importance of methylated CHH context for plant environmental responses and developmental regulation. Our study also revealed that the number of hypermethylated DMRs is higher than that of hypomethylated DMRs in RBSDV-infected rice. This is in agreement with other rice cultivars under abiotic stresses such as drought, salinity and heavy metal stresses [41], indicating that hypermethylation might be more important for rice in the differential stress responses.

To uncover the correlation between promoter methylation status and the level of gene expression in RBSDV-infected rice plants, RNA-Seq was performed to analyze DEGs in relation to DMGs that overlapped with the promoter regions. Cross analysis identified 173 genes (87 hyper-up, 15 hypo-down, 29 hypo-up and 42 hyper-down) with significant difference in RBSDV-infected rice plants compared to the healthy ones. Many of the genes were found to be involved in plant-pathogen interaction, plant hormone signal transduction and several other pathways, suggesting that RBSDV-induced DNA methylation is closely associated with specific genes contributing to plant defense against various pathogens. An innate immune system that plants use to counteract viral infection is broadly divided into pathogen-associated molecular pattern (PAMP)-triggered immunity (PTI) and effector-triggered immunity (ETI) [43]. Activation of MAP kinases (MAPKs) is one of the earliest signaling events that occur in both PTI and ETI [44]. The nucleotide-binding leucine-rich repeat (NB-LRR) domain-containing resistance proteins are employed by plants to recognize viral effectors and activate ETI [45]. *LOC_Os06g17970* and *LOC_Os01g45620* belonging to plant-pathogen interaction pathway were hypermethylated and down-regulated under RBSDV stress. *LOC_Os01g45620* is predicted to encode a MAP kinase with unknown functions. *OsMAPK5b* was reported to suppress salicylic acid-mediated systemic acquired resistance (SAR) [46], a plant immunity against many types of pathogens, suggesting that LOC_*Os*01g45620 may influence infection of RBSDV by regulating SAR. *LOC_Os06g17970* is a NBS-LRR disease resistance protein-like gene whose homolog in *Nicotiana tabacum* confers resistance to tobacco mosaic virus (TMV) [47]. Thus, *LOC_Os01g45620* and *LOC_Os06g17970* might be new target genes involved in regulating plant responses to a variety of pathogen infection. Phytohormones, such as JA, salicylic acid (SA), ABA, auxin and BRs, are small molecules produced within plants that govern plant defense [44]. Previous studies have shown that auxin signaling play a negative role in the defense response against RBSDV infection. Overexpression of the auxin signaling repressors *OsIAA20* and *OsIAA31* has been demonstrated to be conducive to RBSDV infection [25]. In this study, *LOC_Os03g43410* and *LOC_Os05g43920* were found to be auxin response factors. *LOC_Os03g43410* was hypomethylated and up-regulated, whereas *LOC_Os05g43920* is completely opposite under RBSDV stress, indicating that their function on rice defense against RBSDV may be totally different. There is little information about the function of *LOC_Os06g46284* (*glycosyl hydrolase, family 31*, putative, expressed) and *LOC_Os06g21820* (*jasmonate O-methyltransferase*, putative, expressed) on plant resistance against pathogens. The negative correlation between gene expression and methylated promoters level indicates that the expression level of selected genes might be regulated by DNA methylation which still requires follow-up studies. Additionally, it is worth noting that we also identified some hypermethylated and up-regulated plant defense genes, such as LOC_Os04g51560 (WRKY68, expressed) and LOC_Os08g10430 (NBS-LRR disease resistance protein, putative, expressed) (Supplementary Data S1). This result indicates that positive regulation of genes by promoter methylation likely play important roles in rice defense to RBSDV.

DNA methylation and DNA demethylation activities can regulate plant DNA methylation dynamically [48]. We analyzed the expression of 16 genes involved in DNA methylation and demethylation including different types of *DMT* family genes, demethylase-coding genes like *ROS1* and *RDR* protein genes by RT-qPCR. *OsDMT702* and *OsDMT707* are genes that play vital roles in maintaining CpG methylation. *OsDMT702* was repressed significantly under RBSDV infection, while it was induced when rice plants were exposed to Cd [27]. *OsDMT707* was up-regulated in this study which was consistent with the result under Cd stress, but was contrary to that under pesticide stress [35]. *OsROS1* was drastically induced by Cd whereas it was down-regulated by RBSDV [27]. These results suggested that the mechanisms of rice DNA methylation in response to biotic stress or abiotic stress were complicated and the increased DNA methylation during RBSDV infection is likely indirectly caused by decreased expression of *OsDMT702*. RNA-dependent RNA polymerases (RDRs) that generate the 24 nt small interfering RNAs (siRNAs) are involved in siRNA-dependent RNA-directed DNA methylation (RdDM) pathway [36]. The expression patterns of RDRs in rice in response to differential stresses undergone a global increase [27], indicating that the RDRs-dependent RNA-directed DNA methylation in rice was conserved and broad-spectrum.

In conclusion, our investigation shows that infection of RBSDV brings about significant changes in transcriptome and methylation levels in rice. Compared to the healthy rice, a great number of differentially methylated regions in CG and non-CG sites were identified in rice under RBSDV stress. We detected 980,913 non-redundant RBSDV-responsive DMRs. The number of hypermethylated genes was much higher than hypomethylated genes. RNA sequencing revealed 1119 DEGs and their combinational analysis filtered 102/71 genes with positive/negative correlation between their promoter methylation status and gene expression level. Our results revealed new target RBSDV-responsive genes that may be modified by DNA methylation and provided an insight into the antiviral mechanism of rice.

## 4. Materials and Methods

### 4.1. Plant Culture and Treatment

RBSDV-infected and virus-free rice NIP were used for the Whole Genome Bisulfite Sequencing (BS-seq) and RNA sequencing. RBSDV were transmitted experimentally to 10-day-old seedlings by the small brown plant hopper (*Laodelphax striatellus*) for 3 days. Rice plants were then grown in a glasshouse with a condition of a 14/10 light/dark cycle under artificial light at 28–30 °C.

### 4.2. DNA Library Construction and Sequencing

Total genomic DNA of 30-day-old rice seedlings was extracted using QIAamp Fast DNA Tissue Kit (Qiagen, Dusseldorf, Germany) following the manufacturer’s procedure. The DNA quality was determined with A260/280 ratios by spectrophotometer. The DNA samples were fragmented using sonication to generate fragments between 100 and 300 bp then subjected to bisulfite conversion. The Accel-NGS Methyl-Seq DNA Library Kit (Swift, MI, USA) was utilized for attaching adapters to single-stranded DNA fragments. The *Extension step* is used to incorporate truncated adapter 1 by a primer extension reaction. The *Ligation step* is used to add the second truncated adapter to the bottom strand only. The *Indexing PCR step* increases yield and incorporates full length adapters. Bead-based SPRI clean-ups are used to remove both oligonucleotides and small fragments, as well as to change enzymatic buffer composition. Finally, we performed the pair-end 2 × 150 bp sequencing on an illumina Hiseq 4000 platform housed in the LC Sciences (Houston, TX, USA).

### 4.3. Bioinformatics Analysis

Firstly, the reads that contained undetermined bases, low quality bases and adapter contamination were removed by using cutadapt [49] and in house perl scripts. FastQC (http://www.bioinformatics.babraham.ac.uk/projects/fastqc/) was used to verify sequence quality, including the Q20, the Q30, and the GC-content of the clean data. Reads that passed quality (Table S1) control were mapped to rice reference genome (https://genome.jgi.doe.gov/portal/pages/dynamicOrganismDownload.jsf?organism=Phytozome) using WALT [50]. After alignment, reads were further deduplicated using samtool [51]. The ratio of the number of reads supporting C (methylated) to that of total reads (methylated and unmethylated) using per scripts in house and MethPipe [52] determined the the DNA methylation level of each cytosine site. Differentially methylated regions (DMRs) were calculated by R package-MethylKit [53] with default parameters (1000 bp slide windows, 500 bp overlap, *p*-value < 0.05).

### 4.4. mRNA Library Constuction, Sequencing and RT-qPCR

Total RNA from 30-day-old RBSDV-infected and healthy rice leaves were separately isolated using the TRIzol Reagent (Invitrogen, Carlsbad, CA, USA) for RNA-Seq. The total RNA quantity and purity were assayed by Bioanalyzer 2100 and RNA 6000 Nano LabChip Kit (Agilent, Santa Clara, CA, USA) with RIN number >7.0. Poly (A) mRNA were isolated from a total of 10 μg RNA with poly-T oligoattached magnetic beads (Invitrogen) and fragmented into small pieces using divalent cations under elevated temperature. The cleaved RNA fragments were reverse-transcribed to create the final cDNA library following the protocol for the mRNA-Seq sample preparation kit (Illumina, San Diego, CA, USA), the average insert size for the paired-end libraries was 300 bp (±50 bp). The libraries were subjected to paired-end sequencing using IlluminaHiseq4000. Raw sequencing reads were assessed for overall quality using FASTQC. Sequencing specific adaptors or low quality bases (Phred score of six consecutive bases below 15, minimum read length of 36 nt) were removed from the datasets. The remaining high-quality reads (Table S2) of samples were aligned to the rice reference genome using HISAT package. After the final transcriptome was generated, the expression levels of all transcripts were estimated though StringTie and Ballgown. The differentially expressed mRNAs and genes were selected with log2 (fold change) >1 or log2 (fold change) <−1 and with statistical significance (*p*-value < 0.05) by R package-Ballgown. The RT-qPCR was performed using the ABI Quantstudio6 Flex (Applied Biosystems, Foster City, CA, USA) with Hieff^®^ qPCR SYBR Green Master Mix (Yeasen, Shanghai, China). Gene-specific primers used for RT-qPCR are listed in Table S3. *Os*Actin was used as an internal control.

### 4.5. Pathway and Network Analyses

The Kyoto Encyclopedia of Genes and Genomes (KEGG) pathway analysis and Gene Ontology (GO) enrichment analysis were performed to determine the pathways and biological functions of DMRs-associated genes (*p*-value < 0.05) and DEGs (|log2foldchange| ≥ 1, *p* < 0.05). The REVIGO web server (http://revigo.irb.hr/) was used for GO annotation. The KEGG Mapper Annotate Sequence tool with the BlastKOALA server is available on the Kyoto Encyclopedia of Genes and Genomes website (https://www.kegg.jp/kegg/tool/annotate_sequence.html).

### 4.6. Methylation-Expression Correlation Analysis

The correlation between methylation and gene expression was determined by comparison of methylation status of DMR-associated genes and their expression level/differential expression measured by RNA-seq. A box-and-whisker plot (boxplot R function) of the differential expression levels of the genes associated with hypo- or hyper-methylated DMRs and all rice genes was generated. The significance of differences was estimated using a two-tailed Wilcoxon rank-sum test (wilcox.test R function) using R programming environment. DNA methylation and gene expression level of each gene was tested for non-zero correlation using Pearson correlation (R function corr.test).

### 4.7. Statistical Analysis

Differences were analyzed using two-tailed Student’s t-test between two samples. A *p*-value ≤0.05 was considered statistically significant. All analyses were performed using ORIGIN 8 software.

## Data Availability

The DNA methylation and RNA-seq data supporting the results have been deposited with NCBI at Gene expression Omnibus (GEO) under series accession number GSE150378.

## Supplementary Materials

The following are available online at https://www.mdpi.com/1422-0067/21/16/5753/s1, Figure S1: Summary of sequencing and mapping, Figure S2: The density profile of methylcytosines in chromosomes of rice. Figure S3: Statistics of the top pathways enriched for 71 genes with negative correlation between methylation level and gene expression identified though cross analysis of DMGs and DEGs, Figure S4: Combinational analysis of genes that changed in DNA methylation and transcriptional expression, Table S1: Output data of bisulfite sequencing (BS-Seq) of virus-free rice seedlings (Mock) and RBSDV-infected rice seedlings (RB), Table S2: Output data of RNA-seq from virus-free rice (Mock) and RBSDV-infected rice (RB) rice libraries, Table S3: Gene-specific primer sequences used for RT-qPCR, Supplementary Data S1 and Data S2 supported for Figure 4 and Figure S4.
