# Supplementary material for: Alterations of Rice (Oryza sativa L.) DNA Methylation Patterns Associated with Gene Expression in Response to Rice Black Streaked Dwarf Virus
Source: Int J Mol Sci. 2020 Aug 11;21(16):5753. doi: 10.3390/ijms21165753 (PMC7570085; doi:10.3390/ijms21165753)
Supplement: Supplementary file 1 [file ijms-21-05753-s001.zip › Supplementary_Material 1.pdf]

Supplementary Table S1. Output data of bisulfite sequencing (BS-Seq) of virus-free rice seedlings (Mock) and RBSDV-infected rice seedlings (RB).

Sample	Mock-1	Mock-2	Mock-3	RB-1	RB-2	RB-3
Raw data read(M)	85333334	84048720	88499894	93403218	90424096	90666668
Raw base(Gb)	12.80	12.61	13.27	14.01	13.56	13.60
Valid data(M)	82416848	81447850	85564338	90367002	87853866	88001962
Valid base(Gb)	12.36	12.22	12.83	13.56	13.18	13.2
Q30%	95.04	95.03	94.89	94.90	95.11	95.17
GC%	29.34	28.94	28.86	29.15	29.36	29.19
non-conversion rate bisulfite libraries	0.27%	0.23%	0.27%	0.26%	0.23%	0.22%
Unique mapped reads(M)	62422933	62557007	65352203	69947464	67604489	68172039
Unique reads mapping rate (%)	75.74	76.81	76.38	77.40	76.95	77.47

Supplementary Table S2. Output data of RNA-seq from virus-free rice (Mock) and RBSDV-infected rice (RB) rice libraries.

Sample	Mock-1	Mock-2	Mock-3	RB-1	RB-2	RB-3
Raw data read(M)	42597440	44769126	44984598	43973720	41487464	43109462
Raw base(Gb)	6.39	6.72	6.75	6.60	6.22	6.47
Valid data read (M)	42338796	44444404	44731262	43728246	41224028	42841086
Valid base(Gb)	6.35	6.67	6.71	6.56	6.18	6.43
Valid ratio(%)	99.39	99.27	99.44	99.44	99.37	99.38
Mapped reads	41035586	43061501	43102888	42209554	39849630	41323482
Aligned ratio(%)	96.92	96.89	96.36	96.53	96.67	96.46
Unique Mapped reads	31735873	33800865	33298937	32421113	30709765	32366056

Supplementary Table S3. Gene-specific primer sequences used for RT-qPCR.

Primer name	Forward (5'-3')	Reverse (5'-3')
OsActin	GAGTATGATGAGTCGGGTCCAG	ACACCAACAATCCCAAACAGA
LOC_Os03g43410	GTGAGGTCGTACAGGAAGAGC	GTCGATCTTCCTCAGGTACGG
LOC_Os06g46284	AACGTGGCTGATCTTGAGGG	GTTCACGTTGATCCCTGGGT
LOC_Os06g21820	GTCTGCACTGGCTCTCTCAG	CGGCCAACGAAGACTAGGAA
LOC_Os09g04890	TCGTTGGGCTCATCTTGCAT	ACGTGCACAAAGAGGGTGAT
LOC_Os05g43920	GCTGATATGCGGGGCTACAT	ATTCTGTGTACGAGCGCCAA
LOC_Os01g45620	TCCGATCTCCACCAGGTCAT	GTCGAAGGACACTCGAGCAA
OsDMT702	GCATCACTCCTGGAGATGGG	TTGACAGCGGCGTAGAACTT
OsDMT707	CGACTGACAGTTGCATCCCT	ACCAGCAGTGCTTTTTGCAG
OsDMT701	GAAGAGACCTCCCCACATGC	CTCGTCGTACGAGAAGGAGC
OsDMT703	GCAAATGGTACAGCTCTGCG	CCTTGAACCTTCACCCCGTT
OsDMT704	ATTCCAGCGCTATATCCGGC	TCTGGATCCCACTCCACGAT
OsDMT705	ACACACGGCAAGGACTACAG	ACCAAACTGCAAGGGGCTTA
OsDMT706	GTGGGCCATCTGGGAATCAA	ACACCTTTTGGAGCTAGGGC
OsDMT708	AAGATGTGGTGGCCCTCTTG	TCCAACCCAGACAAGGTTCC
OsDMT709	GGAGTTAGCAGGACAGCGAG	CCAACTCCTCAGCAACGTCA
OsDMT710	GGATCACCGTGCCACAACTA	CCTCCTTTCGTGAGAGAGCC
OsROS1	ATGTGTGAGGCTTGGATGGG	TGCACTCAGCTCTCATTGGG
OsRdRp1	AGAGCTGTTGTTGTGTGGCT	GCTCGAATGAACACTTGCCC
OsRdRp2	ATGACGGGAAAACAGCCACA	AATAGAGGTCGCCGTCCAAA
OsRdRp3a	CGGCAGCTGATAGCTGGTTA	GCCCATGAAGTGGGGGTATT
OsRdRp3b	CGTGCTACGGCAGCTACTAA	AAGGACTGCGGTCCATGAAG
OsRdRp6	AGAAGGCACTGAATCGGGTG	GTTCAGCACCTGCATACCCT

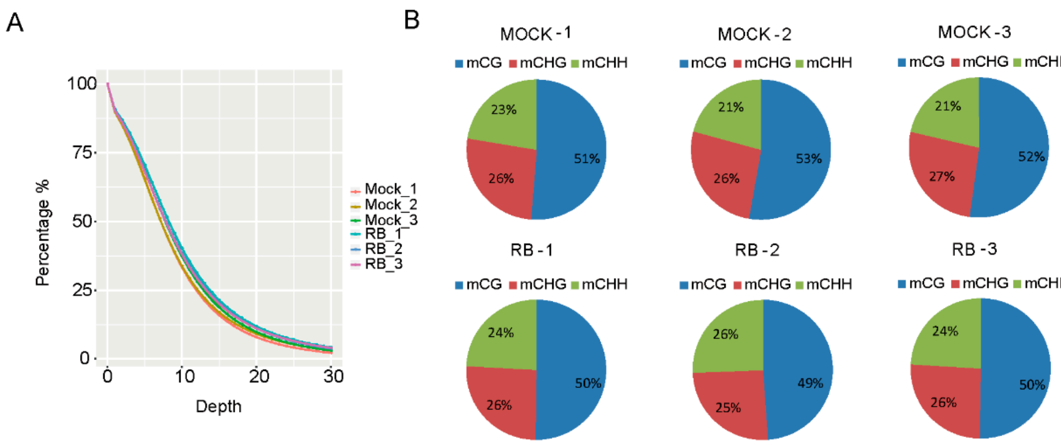

Supplementary Figure S1. Summary of sequencing and mapping. **(A)** The methylcytosine cumulative distribution of effective sequencing depth. The y axis indicates the cumulative methylcytosine distribution of virus-free and RBSDV-infected rice under each effective sequencing. **(B)** Percentage of methylated cytosines identified for each sequence context in rice.

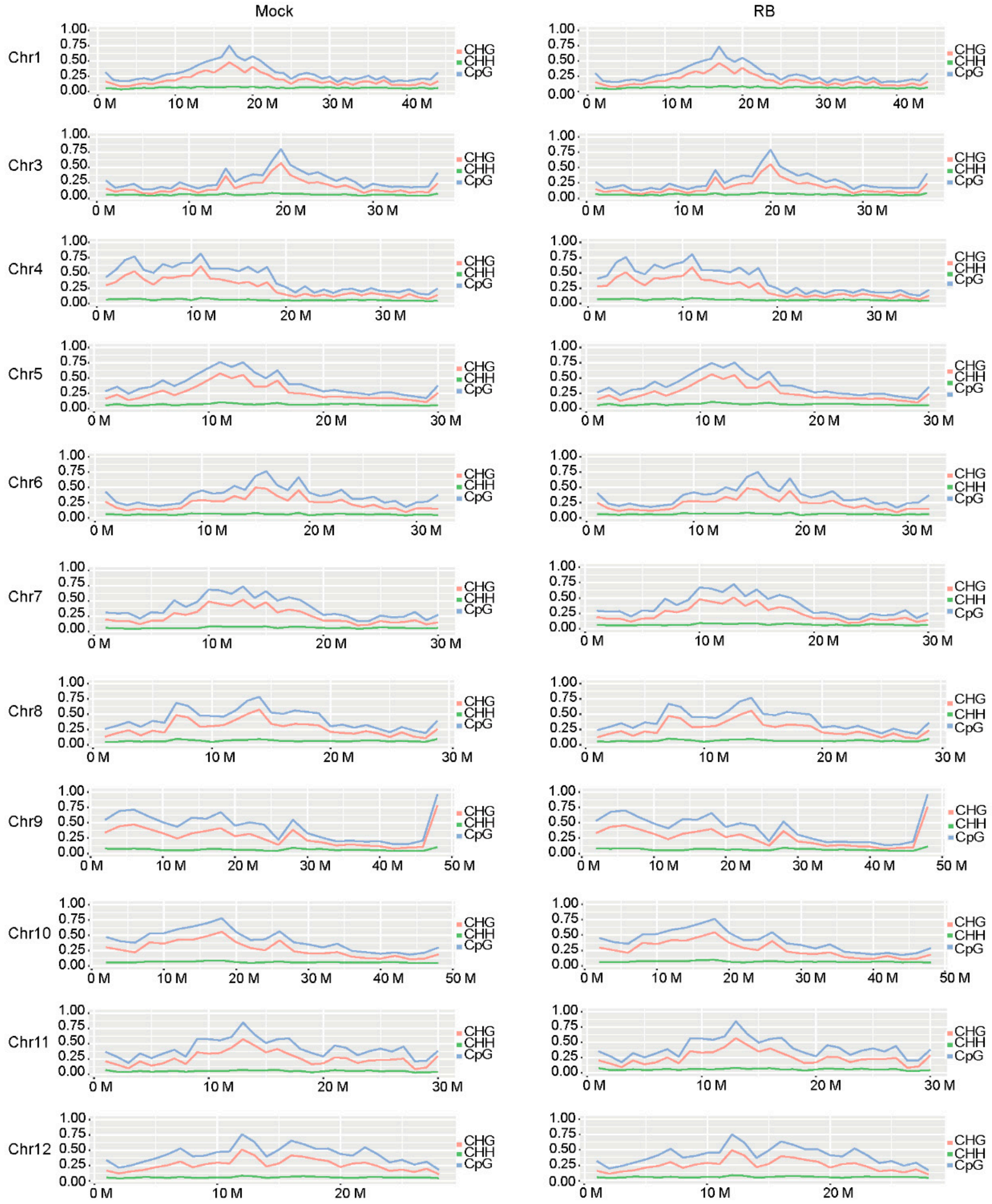

Supplementary Figure S2. The density profile of methylcytosines in chromosomes of rice. Smoothed lines represent the methylation level (CHG, CHH and CpG) density in each context in Mock (virus-free) and RB (RBSDV-infected) rice.

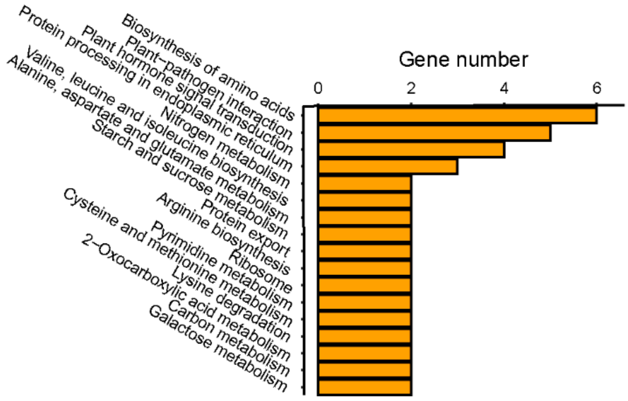

Supplementary Figure S3. Statistics of the top pathways enriched for 71 genes with negative correlation between methylation level and gene expression identified though cross analysis of DMGs and DEGs.

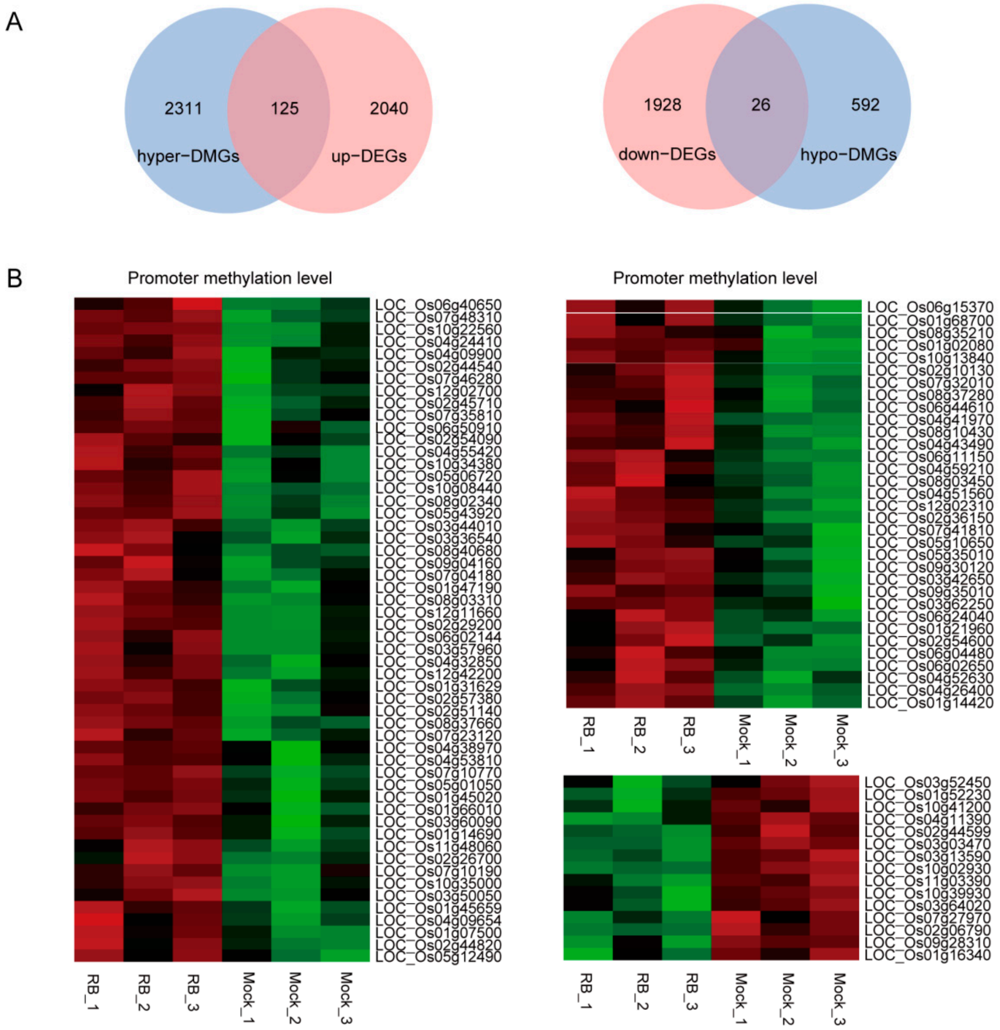

Supplementary Figure S4. Combinational analysis of genes that changed in DNA methylation and transcriptional expression. (A) Venn diagrams display the positive association between DNA methylation region (DMR)-associated genes (*P*<0.05) and differentially expressed genes (*P*<0.05) induced by RBSDV. (B) Heatmap represents the methylation levels of 102 RBSDV-responsive genes.
